# Interaction with Serum Albumin As a Factor of the Photodynamic Efficacy of Novel Bacteriopurpurinimide Derivatives

**Published:** 2015

**Authors:** Akimova Akimova, G. N. Rychkov, M. A. Grin, N. A. Filippova, G. V. Golovina, N. A. Durandin, A. M. Vinogradov, T. A. Kokrashvili, A. F. Mironov, A. A. Shtil, V. A. Kuzmin

**Affiliations:** N.M. Emanuel Institute of Biochemical Physics, Kosygina Str., 4, Moscow, 119334, Russia; Petersburg Nuclear Physics Institute, Orlova Roscha, Gatchina, Leningrad district, 188300, Russia; St.Petersburg State Polytechnical University, Politekhnicheskaya Str., 29, St. Petersburg, 195251, Russia; M.V. Lomonosov Moscow State University of Fine Chemical Technologies, Prospekt Vernadskogo, 86, Moscow, 119571, Russia; N.N. Blokhin Russian Cancer Research Center, Kashirskoe Shosse, 24, Moscow, 115478, Russia; Georgian Technical University, Kostava Str., 77, Tbilisi, 0175, Georgia

**Keywords:** photosensitizers, albumin, association constant, photodynamic therapy, cancer, necrosis

## Abstract

Optimization of the chemical structure of antitumor photosensitizers (PSs) is aimed at increasing their affinity to a transport protein, albumin and irreversible light-induced tumor cell damage. Bacteriopurpurinimide derivatives are promising PSs thanks to their ability to absorb light in the near infrared spectral region. Using spectrophotometry, we show that two new bacteriopurpurinimide derivatives with different substituents at the N atoms of the imide exocycle and the pyrrole ring A are capable of forming non-covalent complexes with human serum albumin (HSA). The association constant (calculated with the Benesi-Hildebrand equation) for N-ethoxybacteriopurpurinimide ethyloxime (compound 1) is higher than that for the methyl ether of methoxybacteriopurpurinimide (compound 2) (1.18×10^5^ M-1 vs. 1.26×10^4^ M^-1^, respectively). Molecular modeling provides details of the atomic interactions between 1 and 2 and amino acid residues in the FA1 binding site of HSA. The ethoxy group stabilizes the position of 1 within this site due to hydrophobic interaction with the protein. The higher affinity of 1 for HSA makes this compound more potent than 2 in photodynamic therapy for cultured human colon carcinoma cells. Photoactivation of 1 and 2 in cells induces rapid (within a few minutes of irradiation) necrosis. This mechanism of cell death may be efficient for eliminating tumors resistant to other therapies.

## INTRODUCTION

The biological effect of exogenous photoactivatable chemical compounds on the cells of prokaryotes and eukaryotes is determined by the formation of reactive species and induction of numerous processes, finally resulting in cell death [1, 2]. This mechanism is used in photodynamic therapy (PDT) for tumors and non-neoplastic and infectious diseases [3-5]. Compounds containing tetrapyrrole macrocycles: porphyrins and their hydrogenated analogues, such as chlorins and bacteriochlorins, are the most frequently used photosensitizers (PSs) in PDT [3].

Structural optimization of PSs, aimed at improving their clinical efficacy, includes the following directions. First, photodamage of deep tissue layers in the lesion should be achieved. For this purpose, bacteriochlorophyll *a *derivatives that absorb light in the longer wavelength range (near the infrared region) of the spectrum are, in particular, used [6-9]. Second, PS should interact with transport proteins, mainly albumins (human serum albumin, HSA), to be efficiently delivered to the pathologic nidus. Increasing the binding affinity can be achieved by introduction of metal cations into the macrocycle and modification of peripheral substituents [10-15]. In addition to the transport function, complexes of HSA with Pd-containing bacteriochlorin act as photocatalytic oxidoreductases, significantly increasing the yield of active oxygen species and the photodynamic effect [2].

Finally, the ability of PSs to cause irreversible photodamage (death) to cells that are resistant to other therapeutic modalities is important. This ability is particularly important in cases where application of other treatment methods is impossible (impossibility of radical surgical treatment, residual tumor after combination therapy, etc.). Photoinduced cell death can occur via the necrotic mechanism. A distinctive feature of this mechanism is primary irreversible damage of the plasma membrane and membrane organelles [5].

The aim of this study was to explore the quantitative parameters characterizing binding of HSA with two bacteriopurpurinimide derivatives that differ from each other by peripheral substituents. These parameters are considered as an efficacy factor of induction of tumor cell photo necrosis.

## EXPERIMENTAL

**Fig. 1:**
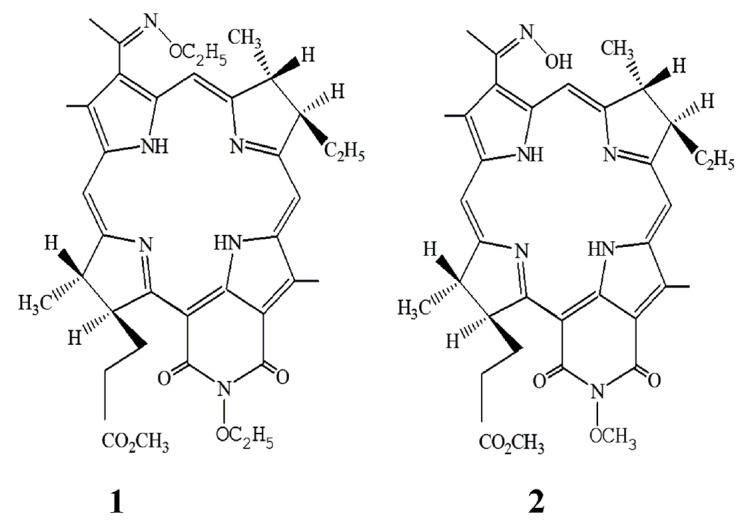
Structures of compounds **1 **and **2**

Bacteriopurpurinimide derivatives with various substituents at the nitrogen atom in the imide exocycle (**1 **– ethoxy group and **2 **– methoxy group) and the pyrrole ring A (**1 **– N-ethoxy group, and **2 **– N-hydroxy group) (*Fig. 1*) were studied. The compounds were prepared by treating bacteriopurpurine with ethoxy amine (**1**) [16] and hydroxylamine (**2**), followed by methylation with diazomethane [17], dissolved in dimethyl sulfoxide (DMSO; Marbiopharm, Russia) to a concentration of 10 mM, and stored at 4 °C. The concentrations of **1** and **2 **were determined based on the known extinction coefficients for chloroform [16, 17].

To study the association, HSA (Sigma Aldrich, USA) was dissolved in a 20 mM phosphate buffer (PB), pH 7.0. The concentrations of **1 **and **2 **were 1 × 10^-5^ M, and the concentration of HSA was 0 – 5 × 10^-4^ M. Stock solutions of compounds **1 **and **2 **in dimethyl sulfoxide (DMSO) were added to PB. The final concentration of DMSO in the tested samples was 1%.

The absorption spectra of the tested solutions were measured on a Shimadzu UVVIS3101PC (Japan) spectrophotometer, using quartz cells (0.4 × 1.0 cm) with an optical path length of 1 cm (spectral slit width was 1 nm). The absorption spectra of the dyes in the presence of HSA were recorded in the range of 380–950 nm.

The binding constants of **1 **and **2 **and HSA were determined based on the change in optical density at the Q-band maximum of a dye associated with HSA, by adding the protein to a solution of **1 **or **2**. The required parameters were calculated with the Benesi-Hildebrand equation [18]:

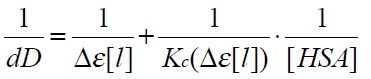



where *dD *is the change in the solution optical density without and with HSA, measured at the absorption peak of the protein complex; *Δε *is the change in the molar extinction coefficient value in the absence and presence of HSA; *K_c_*is the binding constant; [*l*] is the concentration of a ligand **1 **or **2**; and [*HSA*] is the HSA concentration.

The photosensitizing activity of **1 **and **2 **was studied in the HCT116 (human colon cancer) cell line. Cells were cultured in Dulbecco’s modified Eagle’s medium supplemented with 5% fetal calf serum, 2 mM *L*-glutamine, 100 μg/mL streptomycin, and 100 U/mL penicillin (PanEco, Russia) at 37 °C in a humidified atmosphere of 5% CO_2_. Cells were plated in 35 mm Petri dishes (2 × 10^4^ cells in 3 mL of culture medium). After 16 h, compound **1 **or **2 **(final concentrations are provided in the Results section) was added and incubation was continued at 37 °C for 30 min. The culture medium was removed, and the cell monolayer was washed out with PB and irradiated with the red light through the 1 cm water filter (saturated aqueous solution of NaNO_2_). A tungsten lamp was used as a light source. The irradiation period was up to 20 min. Cultures irradiated in the absence of **1 **or **2, **as well as cells loaded with these compounds but not subjected to irradiation (dark exposure), were used as the control. The integrity of the cells was evaluated by the trypan blue exclusion test.

For the purpose of an electron microscopy analysis, HCT116 line cells were plated onto 60-mm Petri dishes (10^5^ cells in 5 mL of culture medium). Addition of **1 **or** 2 **and irradiation of the cells were carried out by the method described above. Next, the cells were detached from the substrate using versene and trypsin and fixed with a 1% glutaraldehyde solution in PB. Samples were analyzed with a Shimadzu electronic microscope (Japan).

Rough spatial molecular models of **1 **and **2 **were generated using the Molsoft ICM version 3.7 [19] and Avogadro [20] software, based on the structure of purpurin *a *taken from the ChemSpider database (www.chemspider.com) (record number: 16736724 of 08.15.2013). Optimization of their structures was carried out using the Gamess US program [21]. The electronic state multiplicity of molecules was taken equal to 1, and molecular charge was neutral. The structure was optimized according to a standard protocol using the quadratic approximation and Huzinaga’s minimal basis set [22]. Self-consistent field wave functions were calculated using the restricted Hartree-Fock method [23].

The spatial structure 1N5U [24] deposited in the Protein Data Bank was used as an initial model of HSA. Molecules of myristic acid, protoporphyrin IX, and water were removed from the structure of HSA prior to flexible docking, and each atom of **1 **and **2 **was charged according to *ab initio *calculations following geometry optimization. Flexible docking was performed using the Molsoft ICM Pro 3.7 package according to the protocol described in detail by the software developers [19, 25, 26]. Docking was run three times, starting from different initial positions and conformations of **1 **and **2 **and HSA. The resulting ensemble of conformations was used to calculate an average binding free energy (*E_C_*) according to the Gibbs-Boltzmann formula:

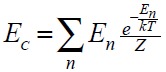



where *Z *is the partition function for the binding free energy of ligands *E_n_*from the ensemble at temperature* T *= 300 K. Only the contributions of electrostatic and hydrophobic components, as well as the entropy contribution of the amino acid side chains of the protein, were taken into account upon calculation of the binding free energy. The electrostatic component was calculated using the REBEL method [27]. According to the recommendations of the software developers, the dielectric constants of HSA, **1**, **2**, and the complexes were set equal to 12.7; the dielectric constant of implicit solvent, was set equal to 78.5. The hydrophobic component of each atom was estimated based on an assumption of its linear proportionality to the atom’s solvent accessible surface area. The atomic salvation parameter was set equal to 0.012 kcal/(mol × A^2^). The loss of configurational entropy of protein amino acid side chains upon binding to **1 **or **2 **was determined using maximal possible entropy read from the program’s residue library [28].

## RESULTS

**Spectrophotometry**

**Fig. 2:**
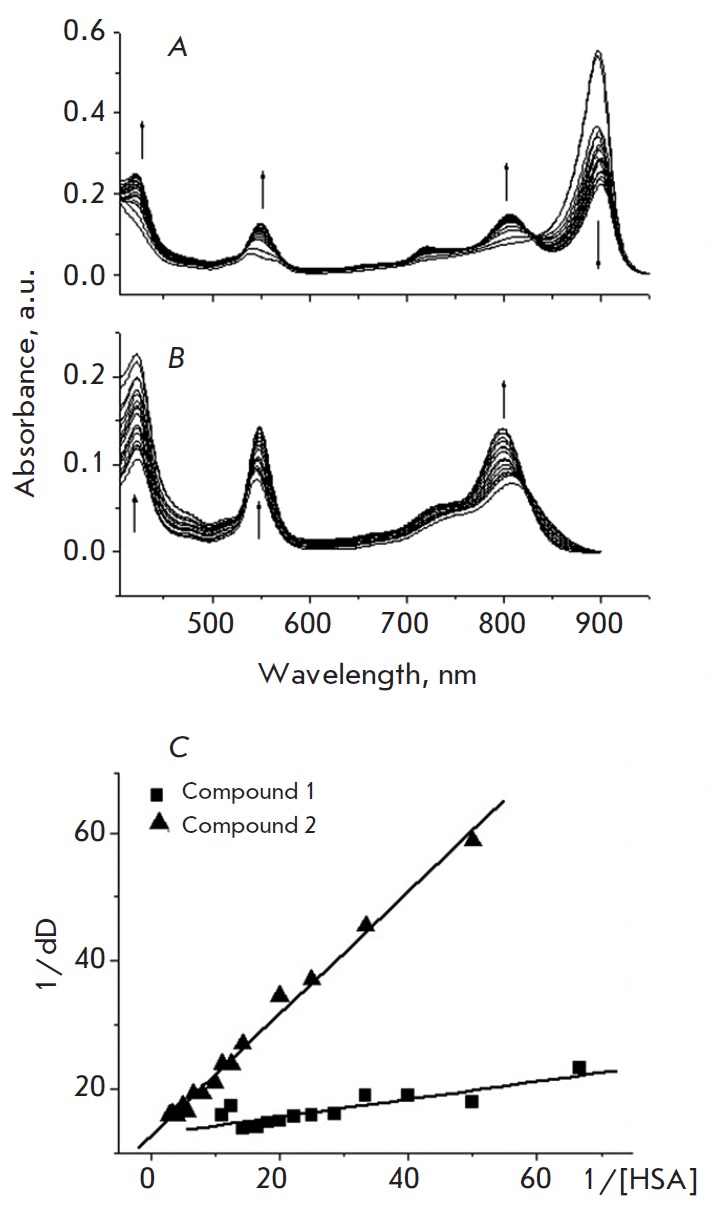
Absorbtion spectra of compounds **1 **(A) and **2 **(B) at different HSA concentrations (20 mM PB, pH 7.0). The Benesi-Hilbedrand plots for complexes of **1 **or **2 **with HSA (C). Arrows indicate the direction of spectral changes upon HSA addition

*Fig. 2* demonstrates the absorption spectra of bacteriopurpurinimide derivatives **1 **and **2 **in the absence and presence of HSA. The absorption bands of **1 **in PB correspond to 539 and 899 nm. The lack of a band at 899 nm in ethanol and chloroform (data not shown) indicates that this band corresponds to the J-aggregate [28]. Compound **2 **in PB is characterized by absorption bands at 421, 550, and 800 nm. The absence of additional bands relative to ethanol and chloroform indicates that compound **2 **does not form J-aggregates in PB.

Transformation of the main absorption bands of both compounds was observed upon addition of HSA. In the case of **1**, this was reflected in the reduction in the intensity of the band at 899 nm, increase in the intensity of the band at 539 nm, and appearance of bands at 419 and 802 nm; the latter probably corresponds to the monomer of **1** (*Fig. 2A*). These results suggest the formation of molecular complexes between **1 **and HSA. The spectra intersect at the isobestic point, indicating equilibrium in the monomer-aggregate system. Therefore, the monomer-aggregate equilibrium shifts towards the monomer as the protein concentration is increased. The obtained result is consistent with the data on the dissociation of aggregates upon complexation of porphyrin derivatives with albumin [29].

*Fig. 2B* shows the absorption spectra of **2 **in the presence of HSA. The optical density of the peaks at 422, 545, and 808 nm increases as the protein concentration rises. A 10-nm hypsochromic shift of the long wavelength maximum is observed. The band with a maximum at 545 nm undergoes a 3.5-nm bathochromic shift to 548.5 nm. The changes in the absorption spectra in the presence of HSA suggest its association with **2**, and the isosbestic point at 835 nm indicates one equilibrium in the monomer-albumin complex and the formation of a stable complex between monomer **2 **and HSA.

The Benesi-Hildebrand plots for **1 **and **2 **and HSA are presented in *Fig. 2C*. The association constant for compound **1 **and HSA is 1.18 × 10^5^ M^-1^, whereas this parameter for **2 **is significantly lower, 1.26 × 10^4^ M-1; i.e., the affinity of compound **1 **to HSA is an order of magnitude higher than that to the complex formed by** 2 **and HSA.

**Molecular modeling**

**Fig. 3:**
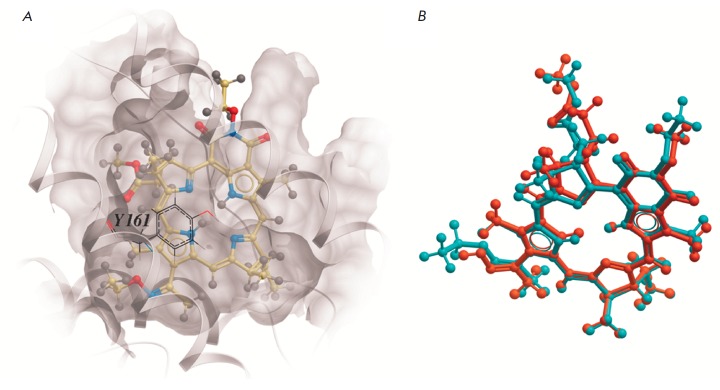
(A) The most probable pose of **1 **in the FA1 binding site of HSA determined by flexible molecular docking. The position of Tyr161 is depicted in the foreground. (B) The most probable conformations of **1 **and **2 **in the binding site are shown. Compound **1 **is shown in blue-green and **2 **in orange. The macrocycles of both compounds have the same spatial arrangement

HSA binding site for heme-like molecules (FA1) is a narrow and quite deep hollow on the surface of the subdomain 1B, formed mainly by hydrophobic amino acid residues [30]. According to the results of flexible docking, compounds **1 **and **2 **are located within the FA1 site in poses similar to that of protoporphyrin IX in the 1N5U crystal structure (Protein Data Bank) (*Fig. 3A*). As these poses have the lowest free energy of binding, *E_C_*, they are the most probable ones. The macrocycle of both compounds effectively “hides” the surface of its hydrophobic groups within the hollow. The hydroxyl group of Tyr161 is located near the macrocycle’s center. However, the macrocycle is shifted by approximately 1 A towards the entrance to the binding site compared to protoporphyrin IX. The results of molecular modeling demonstrate that unlike **1** the conformational diversity of **2 **in the FA1 binding site of HSA is wider. However, the conformations of both compounds having the highest value of the scoring function are almost match (*Fig. 3B*).

**Fig. 4:**
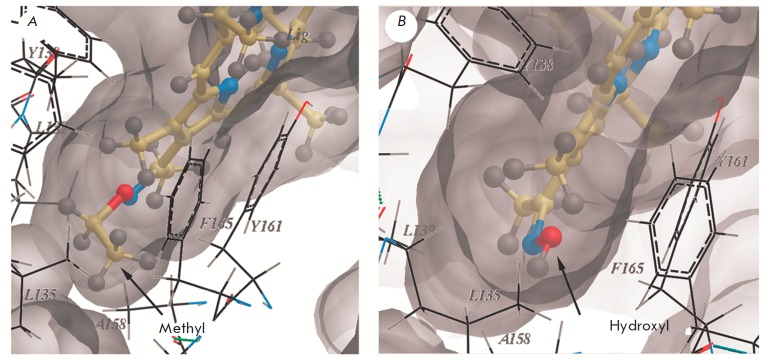
Poses of **1 **and **2 **in the FA1 binding site. The poses of compounds with the maximum scoring function value are shown. The FA1 binding site is displayed as a grey molecular surface. The compounds are presented as a ball-and-stick model. Carbons are shown in beige, hydrogens in grey, nitrogens in blue, and oxygens in red. The non-polar methyl group of **1 **(A) and the polar hydroxyl group of **2 **(B) occur in the hydrophobic microenvironment

Importantly, in this conformation, the hydroxyl group of the pyrrole ring A of compound **2 **occurs in the hydrophobic environment formed by Leu135, Leu139, and Ala168 residues and an aliphatic part of Tyr161 within the binding site (*Fig. 4B*), losing the energetically favorable hydrogen bond. Therefore, it is unlikely that this conformation will be realized in the interaction between **2 **and HSA. In turn, the ethoxy group of the pyrrole ring of **1 **forms an energetically favorable tight hydrophobic contact with HSA in this locus (*Fig. 4A*). The average binding free energy to HSA is –10.5 kcal/ mol for compound **1 **and –9.3 kcal/mol for compound **2** (excluding the indicated conformations). These values correlate well with the association constants obtained experimentally for compounds **1, 2 **and HSA (*Fig. 2*).

**Photodynamic activity in cell culture**

**Fig. 5:**
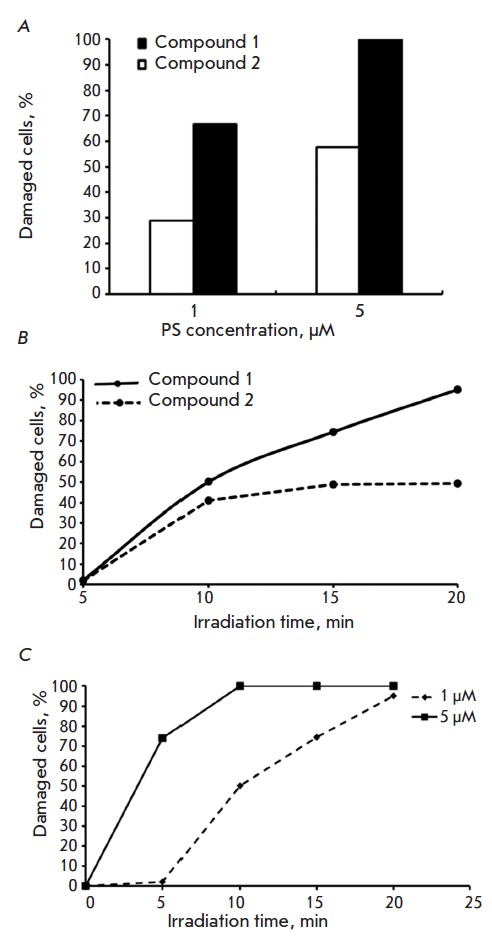
Dependence of HCT116 cell photodamage on the PS concentration and irradiation time. (A) 15 min irradiation; (B) 1 µM **1 **or **2**; (C) compound **1**

Without irradiation, compounds **1 **and **2 **at concentrations of up to 50 μM caused no death of HCT116 line cells under continuous exposure for 72 h. On the contrary, the photosensitizing ability of **1 **and **2 **was high: the micromolar concentrations of **1 **or **2 **were sufficient to induce cell damage. After 15 min of irradiation of cells treated with either of the two tested compounds, the fraction of damaged cells amounted to 100% for **1** and 57.8% for **2** (*Fig. 5A*). After incubation with 1 μM of each PS, the fraction of dead cells increased as the irradiation time was elongated up to 20 min (for **1**), whereas the percentage of dead cells in the case of compound** 2 **did not increase after irradiation for 10–15 min (*Fig. 5B*). Almost complete loss of the culture was observed after 10 min of exposure to light after incubation of the cells with 5 μM of compound **1** (*Fig. 5C*).

**Electron microscopy**

**Fig. 6:**
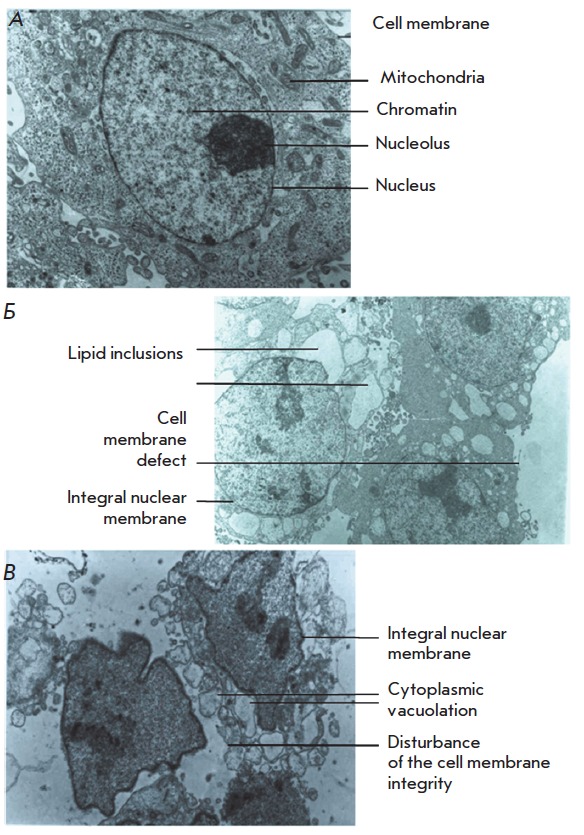
Ultrastructural signs of HCT116 cell photodamage. (A) control cells (irradiated in the absence of 1); (B) 10 min irradiation; (C) 20 min irradiation. In (B) and (C) cases, cells were loaded with 1 µM 1 prior to irradiation. 5,000× magnification

For the purpose of a detailed investigation of the cell death mechanism, we analyzed the ultrastructure of dying cells by transmission electron microscopy. *Fig. 6 A-C*presents the results of electron microscopy of HCT116 cells irradiated in the absence of PS (control) or after incubation with **1**. In the control cells (*Fig. 6A*), the cell membranes formed microvilli, which are typical of the intestinal epithelium, at the free surfaces. Mitochondria, cisterns of the endoplasmic reticulum, ribosomes, and vesicles of the Golgi complex were observed in the cytoplasm. Chromatin was diffusely distributed over the nucleus, with denser clusters located mainly on the periphery. The nuclei were round with shallow invaginations of the nuclear membrane.

After 10 min of irradiation of cells loaded with compound **1**, swelling of mitochondria and a reduction in their matrix density were observed (*Fig. 6B*). Mitochondria with damaged cristae and a “washed out” matrix, lipid droplets, and a small expansion of the endoplasmic reticulum cysternae appeared there. Most cells had an irregular shape due outgrowths on the cell membranes. The integrity of the cell membranes was retained. The amount of chromatin in the nuclei was decreased, and the regions of dense fibrillar component in the nucleoli were increased. After 20 min of irradiation, the number of lysosomes and lipid inclusions in the cytoplasm was increased and the amount of chromatin in the nuclei was decreased. A significant number of cells were destroyed (*Fig. 6C*).

## DISCUSSION

The modification of peripheral substituents in the bacteriopurpurinimide molecule was found to alter significantly the photodynamic efficacy of PS. The bacteriopurpurinimide derivative with ethoxy groups at nitrogen atoms in the exocycle and pyrrole ring A (compound **1**) forms stronger complexes with the transport protein HSA. These results were obtained experimentally and confirmed by molecular modeling of the PS-HSA complexes. Reduced affinity of compound** 2 **for HSA is caused by occurrence of its oxime hydroxyl group in the hydrophobic environment upon binding within the FA1 site. In this case the energetically favorable hydrogen bond with water is lost, which weakens binding to the protein. Conversely, the ethoxy group of compound **1 **promotes stronger binding to the protein due to hydrophobic interactions.

Compounds **1 **and **2 **appear to be highly active PSs: micromolar concentrations and brief incubation were sufficient to induce cell death. It is important that a higher association constant for compound **1 **and HSA corresponded to a higher photoactivity of this PS in the cell culture. The effect of peripheral substituents on photoactivity parameters such as accumulation and distribution of PS in cells, the ability to generate reactive species (yield of singlet oxygen or oxygen radicals) should be evaluated.

We suppose that the increased affinity of **1 **for HSA leads to a high yield of active oxygen species during photoactivation. These are the key metabolites for the processes of photodamage to biomacromolecules. The non-covalent **1**-HSA complex can act as a light-activated oxidoreductase and repeatedly catalyze electron transfer from a PS molecule in the triplet state to molecular O2, boosting the formation of active oxygen species. The mechanism according to which PS in the excited triplet state can directly interact with a substrate and/or solvent through electron or proton transfer, was described previously [2].

The high photoactivity of **1 **and **2 **results in necrosis of tumor cells – primary damage to the cell membrane. Photo necrosis was detected in the first few minutes of exposure to light and accompanied by pronounced and irreversible damage to cell structures. Such a damage was idenified in the cytoplasm, whereas the nucleus retained its structure. A similar photo necrosis pattern was observed upon activation of the membrane-active boronated chlorin derivative e6 [5]. These features differentiate** 1 **and **2 **from other PSs that cause photo-induced cell death through other mechanisms (apoptosis and autophagy) [31-34]. We believe that rapid death of tumor cells as a result of PDT is desirable in clinical situations, especially in order to eliminate tumors with primary or acquired drug resistance. However, it is necessary to assess the significance of possible immunological reactions in response to necrosis-inducing PDT.

In this work, the necessity to optimize long wavelength (infrared) PSs for PDT is demonstrated. The optimization criteria includes increased affinity to the transport protein HSA and the ability to provoke photo necrosis. . Indeed, the chemical modification of bacteriopurpurinimide enables the production of a compound with increased affinity for HSA and the ability to cause irreversible photodamage to tumor cells. These features, as well as the lack of dark cytotoxicity and sufficient solubility in aqueous media (at least in the range of concentrations required to induce photo necrosis), make new bacteriopurpurine derivatives promising for further research.

## Abbreviations

DMSO: dimethyl sulfoxide
PB: phosphate buffer
PDT: photodynamic therapy
PS: photosensitizer
HSA: human serum albumin

## References

[1] Phillips D. (1997). Int. Rev. J..

[2] Ashur I., Goldschmidt R., Pinkas I., Salomon I., Szewczyk G., Sarna T., Scherz A. (2009). J. Phys. Chem. A..

[3] Josefsen L.B., Boyle R.W. (2012). Theranostics..

[4] Chen Y., Li G., Pandey R.K. (2004). Curr. Org. Chem..

[5] Moisenovich M.M., Ol’shevskaya V.A., Rokitskaya T.I., Ramonova A.A., Nikitina R.G., Savchenko A.N., Tatarskiy V.V., Kaplan M.A., Kalinin V.N., Kotova E.A. (2010). PLoS ONE.2010. V. 5,.

[6] Grin M.A., Mironov A.F., Shtil A.A. (2008). Anti-Cancer Agents Med. Chem..

[7] Oertel M.l., Schastak S.I., Tannapfel A., Hermann R., Tannapfel A., Hermann R., Sack U., Mossner J., Berr F. (2003). J. Photochem. Photobiol. B: Biology..

[8] Dąbrowski J.M., Arnaut L.G., Pereira M.M., Urbańska K., Simões S., Stochel G., Cortes L. (2012). Free Rad. Biol. Med..

[9] Meerovich I.G.., Grin M.A., Tsiprovskiy A.G., Meerovich G.A., Oborotova N.A., Loschenov V.B., Baryshnikov A.Y., Mironov A.F. (2007). Russian Biotherapeutic J..

[10] Ol’shevskaya V.A., Nikitina R.G., Guiul’malieva M.A., Zaitsev A.V., Luzgina V.N., Kononova E.G., Ivanov O.G., Burmistrova N.V., Kaplan M.F., Kalinin V.N. (2006). Org. Biomol. Chem..

[11] Ol’shevskaya V.A., Nikitina R.G., Savchenko A.N., Malshakova M.V., Vinogradov A.M., Golovina G.V., Belykh D. V., Kutchin A.V., Kaplan M.A., Kalinin V.N. (2009). Bioorg. Med. Chem..

[12] Ol’shevskaya V.A., Savchenko A.N., Zaitsev A. V., Kononova E. G., Petrovskii P.V., Ramonova A.A., Tatarskiy V.V. Jr., Moisenovich M.M., Kalinin V.N., Shtil A.A. (2009). J. Organometal. Chem..

[13] Pshenkina N.N. (2011). Med. Academ. J..

[14] Sharman W.M., van Lier J.E., Allen C.M. (2004). Adv. Drug Delivery Rev..

[15] Tsuchida T., Zheng G., Pandey R.K., Potter W.R., Bellnier D.A., Henderson B.W., Kato H., Dougherty T.J. (1997). Photochem. Photobiol..

[16] Mironov A.F., Grin M.A., Tsiprovskiy A.G., Meerovich G.A., Meerovish I.G., Oborotova N.A., Treshalina E.M., Loschenov V.B., Baryshnikov A.Y., Tsigankov A.A. (2011). Patent of Russia № 2411943. Bull. № 29..

[17] Mironov A.F., Grin M.A., Tsiprovskiy A.G. (2002). J. Porph. Phthalocyan..

[18] Benesi H.A., Hildebrant J.H. (1949). J. Am. Chem. Soc..

[19] Abagyan R., Totrov M., Kuznetsov D. (1994). J. Comput. Chem..

[20] Hanwell M.D., Curtis D.E., Lonie D.C., Vandermeersch T., Zurek E., Hutchison G.R. (2012). J. Chem. Inform..

[21] Schmidt M.W., Baldridge K.K., Boatz J.A., Elbert S.T., Gordon M.S., Jensen J.H., Koseki S., Matsunaga N., Nguyen K.A., Su S., Windus T.L., Dupuis M., Montgomery J.A. (1993). J. Comput. Chem..

[22] Huzinaga S., Andzelm J., Klobukowski M., Radzio-Andzelm E., Sakai Y., Tatewaki H. (1984). Gaussian Basis Sets for Molecular Calculations.Amsterdam: Elsevier.

[23] Roothaan C.C.J. (1951). Rev. Modern Phys..

[24] Wardell M., Wang Z., Ho J.X., Robert J., Ruker F., Ruble J., Carter D.C. (2002). Biochem. Biophys. Res. Commun..

[25] Fernández-Recio J., Totrov M., Abagyan R. (2003). Proteins: Structure, Function, and Bioinformatics..

[26] Totrov M. and Abagyan R. (1997). Proteins..

[27] Totrov M., Abagyan R. (2001). Peptide Sci..

[28] Eisfeld A., Briggs J.S. (2006). Chem. Phys.

[29] Yao-Bing Y., Wang Y.N., Ma J.B. (2006). Spectrochim. Acta.

[30] Ascenzi P., Fasano M. (2009). IUBMB Life..

[31] Garg A.D., Bose M., Ahmed M.I., Bonass W.A., Wood S.R. (2012). PLoS ONE..

[32] Chin W.W., Heng P.W., Bhuvaneswari R., Lau W.K., Olivo M. (2006). Photochem..

[33] Calin M.A., Paraska S.V. (2006). J. Optoelectron. Adv. Mat..

[34] Evans C.L., Abu-Yousif Adnan O., Jin P. Yong., Klein O.J., Celli J.P., Rizvi I., Zheng X., Hasan T. (2011). PLoS ONE..

